# Everybody's Page

**Published:** 1891-05-16

**Authors:** 


### Everybody's Page.

Those who object to the ousting of the beautiful from the few public thoroughfares in our towns which can boast of its possession will rejoice to hear that the London Tramways Bill, to which we have referred previously, and which, if successful, was destined to destroy the beauty of the Embankment, has been thrown out of the House of Commons —so the one unique part of London is not yet to be vulgarised.

We feel a sympathy with the cabby on strike. After all nobody has to stand wet and wind all hours of the day and night as much as the cabmen. The sums they pay for their cabs are large, and the chances of profits, excepting in the crush of the season, very uncertain. Either fares must be raised or the price for the cab lowered, and in these days of cheap railways and omnibus fares the former solution will not help the difficulty. The poor cabbies are, like many other folk, the victims of an overcrowded labour market.

Judge John P. Altgeld appears to be very diffident as to the stability of the American commonwealth, and his diffidence arises from his innate objection to pretence and humbug, no matter whether found in high stations or low. He writes in a melancholy strain to the *Chicago Globe*, that if the American people ever reach a point where they must put robes on their Judges or any other officers in order to have the highest respect for them, then republican institutions will be at an end in America. It is true that no robe ever enlarged a man's brain, ripened his wisdom, cleared his judgment, strengthened his purpose, or fortified his honesty. If he is a little man without a robe he is contemptible in a robe. But it is not necessarily true that if a man is large without a robe he is simply ludicrous in one. Judge John P. Altgeld pays his countrymen a doubtful compliment when he fears that they are so simple-minded as to be inspired with extra respect by such an aristocratic subterfuge and bolstering of weak intellects as a Judge's gown. How hopelessly far off true democracy the benighted Britisher must still be.

It has during recent years been complained, with more or less reason, that in many markets throughout the world the Germans have outstripped us in energy and enterprise. Herr Bolle has certainly given proof of the enterprise of his countrymen by the establishment in Berlin of a model dairy, which has proved such a success as a commercial undertaking as to make a contributor to the *Nineteenth Century* ask very pertinently why he has not found imitators in our large towns. Wholesome and cheap milk is far too conspicuous by its absence from the dietary of our poorer classes. From Herr Bolle's dairy milk is supplied at 2½d. per 1¾ pints in locked cans, placed inside a cart consisting of a huge metal box on wheels. When the box is closed the milk cannot be tampered with, and on the outside, just above the holes where the taps appear, the nature and contents, and the price of each can, are legibly painted. Every driver of a cart is told off to supply a certain portion of the town, and is given a map of the district he has to serve, on which are marked the corners of the streets where he has to stop and sell milk to all who wish to buy. The success of the dairy is shown by the fact that exactly ten years ago it was started with three carts, whereas now 107 carts are used, and 600 men employed. The adoption of such a system in our large towns would be one of the best engines in the hands of the advocates of temperance in their crusade against the poor man's greatest enemy.

The day may yet come when the rose will bloom in the desert, if the report which comes from Paris is true, that a large body of water has been discovered at El Golea, in the Sahara desert, about 120 feet below the surface. When perfect access to the water is attained, the yield, which now amounts to 40 gallons of water a minute, will no doubt be much greater. It is asserted that water in any quantity has never before been found at such a slight depth below the surface in the Sahara.

Though during the past few years there has been an undoubted extension of the free library movement in London generally, the ratepayers of Paddington appear to have set their faces steadily against the adoption of the Free Public Libraries Acts. The recent poll has once more shown in a marked manner the adverse feeling of the inhabitants, of whom only 1,590 have been found ready to give a favourable vote. There are many who are sceptical as to the great advantages to be derived from these institutions; their opinions may be heretical, but if public sentiment is undergoing any change, it looks very much as if it were going slightly in favour of the heretics.

The Earl of Meath has once more called attention to the ever-increasing danger to the metropolis arising from the existence in its midst of large cemeteries. Seeing how hard a prejudice dies in this country, the danger cannot too often be insisted on. The West Brompton Cemetery lies in the midst of a large population, and in its 38 acres contains no fewer than 150,000 bodies, whereas in the Tower Hamlets Cemetery, of 17 acres, 250,000 interments have taken place. Yet with the clear knowledge before them that the presence of such grounds in the heart of populous places must add to the impurities of the air, the great majority of people shudder at the suggestion of the general introduction of cremation as though they were being asked to commit a deliberate crime.

The parson sees men at their best, the lawyer at their worst, but the doctor as they really are. Mr. Edward Berdoe claims this actual knowledge of the ways of the working people of the slums in London, and on the strength of this knowledge has written a short, clear, convincing article on "death-clubs" in this month's "Nineteenth Century." Dr. Berdoe, as an East-end physician, testifies to the devotion of slum mothers. He has found them ever ready to call in a doctor "just for safety like," and in all his experience it is the mother who has suffered want rather that the child. "It is a common thing after the long illness of a member of a poor household for the mother to break down from scanty feeding and anxious watching"; and, again, "the bishops, clergymen, coroners, doctors, and other worthy persons who believe that a child-insurer is a probable child-murderer are led away by their own theories." We have heard much of child insurance lately and its evils, but there must be two sides to the question, and here is the evidence of a man who knows the slums well. While not defending the "beery self-indulgent pauper male parent," Dr. Berdoe, quoting Coleridge, says: "A mother is the holiest thing alive, and I should endorse the sentiment even had I no other experience wherewith to illustrate it than that gleaned from my East London parish work." This is food for reflection for those who believe that hearts and human kindness are monopolies of the "upper classes."

